# Parthenogenesis affects interspecific competition between *Megalurothrips usitatus* and *Frankliniella intonsa* (Thysanoptera: Thripidae) in changing environment: evidence from life table study

**DOI:** 10.1093/jee/toad180

**Published:** 2023-10-04

**Authors:** Ling-Hang Guo, Sheng-Yong Wu, Run-Na Gong, Liang-De Tang

**Affiliations:** National Key Laboratory of Green Pesticide, Key Laboratory of Green Pesticide and Agricultural Bioengineering, Ministry of Education, Center for R&D of Fine Chemicals of Guizhou University, Guiyang 550025, China; School of Plant Protection, Hainan University, Haikou 570228, China; State Key Laboratory for Biology of Plant Diseases and Insect Pests, Institute of Plant Protection, Chinese Academy of Agricultural Sciences, Beijing 100193, China; National Key Laboratory of Green Pesticide, Key Laboratory of Green Pesticide and Agricultural Bioengineering, Ministry of Education, Center for R&D of Fine Chemicals of Guizhou University, Guiyang 550025, China; National Key Laboratory of Green Pesticide, Key Laboratory of Green Pesticide and Agricultural Bioengineering, Ministry of Education, Center for R&D of Fine Chemicals of Guizhou University, Guiyang 550025, China

**Keywords:** thrips, parthenogenesis, cowpea, age–stage life table, temperature

## Abstract

The thrips *Megalurothrips usitatus* Bagnall and *Frankliniella intonsa* Trybom (Thysanoptera: Thripidae) are important pests in cowpea-growing areas of south China. Parthenogenesis is an important reproductive form of thysanopterans, and plays an important role in maintaining population growth. To understand the developmental and reproductive characteristics of these 2 thrips species during parthenogenesis, we compared the age–stage life tables of *M. usitatus* and *F. intonsa* on cowpea pods under natural regimes during the summer and winter. The results showed that the total preadult period and total preoviposition period of *M. usitatus* were significantly longer than those of *F. intonsa* in both seasons. Moreover, longevity of adult *M. usitatus* (29.53 days) was shorter compared with adult *F. intonsa* (34.00 days) in summer, whereas higher fecundity (220.8 eggs/female) and more oviposition days (37.83 days) were observed in *M. usitatus* compared with *F. intonsa* in winter (fecundity = 179.83 eggs/female, oviposition days = 33.03 days). The net and gross reproductive rates of *M. usitatus* were significantly greater than those of *F. intonsa* during winter. In addition, the intrinsic and finite rates of increase of *M. usitatus* were significantly lower than those of *F. intonsa*, and the mean generation time of *M. usitatus* was significantly longer than that of *F. intonsa* both in summer and winter. These results indicated that parthenogenesis has species specificity among thrips, which in turn affects population development, especially under changing environments.

## Introduction

*Megalurothrips usitatus* Bagnall and *Frankliniella intonsa* Trybom (Thysanoptera: Thripidae) are important pests of legume crops. Among them, cowpea, *Vigna unguiculata* (L.) Walp (Fabales: Fabaceae) is the main host plant of *M. usitatus*. *Megalurothrips usitatus* can cause damage throughout the vegetative period of cowpea by feeding and laying eggs, especially during flowering/fruiting periods (Tang et al. 2022, 2023). Damaged flower buds/flowers lead to premature abscission, whereas damage to pods causes pericarp scabbing (Ngakou et al. 2008, Fan et al. 2013, Tang et al. 2015a). *Megalurothrips usitatus* also can cause serious damage by spreading plant viruses (Wu et al. 2018, Zhang et al. 2021). *Frankliniella intonsa* is another important pest on cowpea in south China (Tang et al. 2015a). It has a wide range of hosts and feeds preferentially on flowers; thus, it mainly damages cowpea during the flowering/fruiting stage (Chang 1995, Lim et al. 2013, Fu et al. 2022). Given their characteristics of small body size, short generation cycle, and high reproductive rate, these 2 species significantly reduce the yield and quality of cowpea, restricting the healthy and sustainable development of cowpea agriculture (Zhang et al. 2008, Sani and Umar 2017).

As a survival and reproductive strategy, parthenogenesis enables insects to survive in adverse environments and makes their widespread distribution possible (O’Woma et al. 2016). It is divided into 2 forms: thelytoky and arrhenotoky (Cai 1989, Normark and Kirkendall 2009, Wang 2011). Thelytoky allows females to produce only daughters and eliminates the need to find or attract a mate, which maximizes the rate of increase (Normark and Kirkendal 2009). Thelytoky represents a radical departure from sexual reproduction and has received more attention. In contrast, in arrhenotoky, individuals that develop from unfertilized eggs are male. For parthenogenetic species, the effect of males on population growth is limited. Therefore, there are few studies on arrhenotoky. The mode of reproduction in Thysanoptera is based on haplodiploidy and the 2 forms of parthenogenesis are also based on this (Kumm and Moritz 2010, Li et al. 2015). *Megalurothrips usitatus* and *F. intonsa* are capable of both bisexual and arrhenotokous parthenogenetic reproduction (Tang et al. 2015b, Fu et al. 2022). Since arrhenotokous parthenogenetic reproduction does not require mating, virgin females can produce haploid male offspring. Although its offspring lack genetic information from male parents, it does not affect their backcrossing with their female parents to maintain the reproduction and size of the population. For example, female *Frankliniella occidentalis* Pergande (Thysanoptera: Thripidae) are more tolerant than males of extreme temperatures (Wang et al. 2014); thus, females can produce numerous male offspring through parthenogenesis under adverse environmental conditions to maintain the male–female balance in the population and subsequently keep the population stable (Ding et al. 2018).

Parthenogenesis is also responsible for the rapid spread of thrips (Jenser and Szénási 2004). In addition, studies of parthenogenesis in some insect species showed that disease resistance was significantly improved between populations (Oldroyd 2007, Hughes et al. 2008). Therefore, parthenogenesis is considered to be an important factor in maintaining the population dynamics of insects, including thrips (Rong et al. 2000, Li et al. 2016). However, parthenogenesis is an important but under-researched factor that may be significantly impacts the agricultural and horticultural industries (Hoffmann et al. 2008).

Life tables are important tools used to study insect population ecology and can be used to determine population parameters of research object, such as survival, development, longevity, and fecundity, in various environments (Chi and Liu 1985, Chi 1988, Huang et al. 2017, Govindan and Hutchison 2020). Age–stage, two-sex life table improve on traditional life tables, which only consider the life process of female insects (Southwood 1978, Chi et al. 2019); the former approach fully considers the instar differentiation of insects and includes all individuals in the population (Chi 1988, Huang and Chi 2012). Previous studies of life tables have focused on sexual reproduction rather than parthenogenesis in thrips, such as *Thrips orientalis* Bagnall (Thysanoptera: Thripidae) (Maisnam et al. 2019), *Scolothrips longicornis* Priesner (Thysanoptera: Thripidae) (Heidarian et al. 2020), and *Heliothrips haemorrhoidalis* Bouche (Thysanoptera: Thripidae) (de Souza et al. 2022). In addition, the set artificial temperature conditions are mainly constant or fluctuating (Bayu et al. 2017, Sun et al. 2019, Wang et al. 2021), rather than natural environmental regimes. *Megalurothrips usitatus* and *F. intonsa*, which are sympatric pests of cowpea in south China and share a common ecological niche. We hypothesized that these 2 closely related thrips species exhibit interspecific competition and even species displacement. Based on life table parameters of bisexual reproduction results indicated that *F. intonsa* has a greater interspecific competition advantage than *M. usitatus* (Tang et al. 2023). Parthenogenesis is an important way to maintain the stability of insect populations, and the use of natural regimes in the laboratory are more similar to the natural environment of insects. In order to further clarify the effect of parthenogenesis on the interspecific competition between these 2 thrips species, in this study, population parameters of *M. usitatus* and *F. intonsa* in parthenogenesis were investigated under natural environmental conditions both in summer and winter by using an age–stage, two-sex life table approach.

## Materials and Methods

### Insects and Experimental Conditions

*Megalurothrips usitatus and F. intonsa* colonies were collected from a cultivated cowpea farm (19°51ʹ56″N; 110°20ʹE38″) in Chengmai country, Hainan Province, China. Thrips were collected from cowpeas planted in net houses without applying any insecticides. Before experiments were initiated, the 2 thrips species were reared on cowpea pods in glass bottles (7 cm in diameter, 11 cm in height) with lids (Tang et al. 2015b). Fresh cowpea pods were cut into approximately 8 cm and placed in glass bottles. The rough hand paper was used as a substrate to pad the bottom of the bottle. Each bottle contained 3~4 pods, and then about 100 pairs of thrips adults were inoculated. The bottles were maintained in natural environmental conditions in an outdoor net room for thrips rearing more than 2 generations.

Experiments were conducted during the summer and winter. The temperature during each experiment was recorded hourly using an intelligent hygrothermograph (LYWSD03MMC, Xiaomi Technology Co., Ltd, Beijing, China). The average daily temperature was calculated from hourly temperature measurements across 24 h, and a temperature diagram was drawn for each season, with an average temperature of 27.3 °C in the summer and 21.6 °C in the winter (Supplementary Fig. S1). The relative humidities of the summer and winter experiments were ~85% and ~80%, respectively. Under natural light, the photoperiods in summer and winter were ~13L:11D h and 11L:13D h, respectively.

### Life Table Study

About 100 pairs female and male adults were placed in a glass bottle (7 cm in diameter, 11 cm in height) for free mating 12 h and then allowed to lay eggs on cowpea pods for 12 h, whereupon the adults were removed. When first-instar nymphs appeared from the pods, 120 were randomly selected and each individual was placed separately into a 5-ml centrifuge tube. The tube was prepared with absorbent paper as the pupation substrate and a cowpea pod (2 cm in length) as food. Absorbent cotton was plugged into the tube mouth to prevent thrips from escaping and to maintain air circulation. Cowpea pods were replaced with fresh ones every 3 days until adult eclosion. After the emergence of adults, virgin females (*n* = 30) were placed separately in individual 1.5-ml centrifuge tubes to allow lay eggs. The cowpea pods were cut into ~1-cm lengths for feeding and were replaced daily until the death of the female thrips. Immature stage development and survival were observed at 12-h intervals until the nymphs became adults or died. Dead individuals of any developmental stage were not included when calculating the average developmental time at a specific stage. The various developmental stages were identified by the method described by the references (van Rijn et al. 1995, Zhang et al. 2007). Given that thrips lay their eggs inside the pod tissue, the time before the nymphs appeared was recorded as the developmental period of the egg, but it was not possible to determine egg mortality (van Rijn et al. 1995, Zhang et al. 2007, Park et al. 2010). In a parallel experiment, the cowpea pods that were removed were then stored individually in a new 1.5-ml centrifuge tube for at least 7 days to observe the number of the first-instar nymphs produced by their mother. The number of first-instar nymphs were used to calculate the daily fecundity of females (Tang et al. 2015b). Durations of preoviposition and oviposition, fecundity and adult longevity were also investigated in both summer and winter regimes.

### Life Table Analysis

Data were analyzed according to the two-sex life table theory (Chi and Liu, 1985, Chi et al., 2022). According to this theory, when constructing the parthenogenetic life table, only the female data are entered; given that it is not possible to distinguish immature male from immature female thrips, the survival rate of the female was not considered (i.e., survival rate = 1) (Zhao et al. 2021). The relevant definitions and formulae used based on this theory are showed in Supplementary Table S1. The TWOSEX-MSChart program was used to calculate these demographic parameters (Chi 2022). The bootstrap technique with 100,000 resamplings was used to estimate the standard errors of these population parameters (Efron and Tibshirani 1993, Chi 2022). Paired bootstrap test in the TWOSEX-MSChart program was used to test the significance of differences in the parameters (*P* ≤ 0.05) (Wei et al. 2020). All figures were created using Sigma Plot 12.0 (Systat Software Inc., San Jose, CA, USA).

## Results

### Life Table Parameters of Both Thrips Species Under Summer and Winter Regimes

The developmental times of the egg, nymphal, pupal, and preadult (immature) stage of parthenogenetic offspring of *F. intonsa* were significantly shorter than those of *M. usitatus* under both summer and winter regimes. Significant differences were found in the total preadult period between *M. usitatus and F. intonsa* females in both summer and winter. Female adult longevity of *F. intonsa* (34.00 days) was significantly longer in summer than that of *M. usitatus* (29.53 days), whereas there was no significant difference between the 2 species in winter. Female total longevity was also not significantly different between the 2 species in either summer or winter. Regardless of whether under a summer or winter regime, *F. intonsa* had a shorter total preoviposition period (8.22 and 15.97 days) compared with *M. usitatus* (9.98 and 18.75 days), respectively. The number of ovipositional days and mean fecundity of *F. intonsa* (33.03 and 179.83 eggs/female, respectively) were significantly lesser than that of *M. usitatus* (37.83 and 220.80 eggs/female, respectively) in winter, whereas there was no significant difference between the species under a summer regime (Table 1).

**Table 1.** Female adult longevity, total longevity, total preoviposition period, oviposition days, and fecundity of parthenogenetic *Megalurothrips usitatus* and *Frankliniella intonsa* reared on cowpea pods under summer and winter regimes

Parameters	Summer (*n* = 30)		*P*-value	Winter (*n* = 30)		*P*-value
	*M. usitatus*	*F. intonsa*		*M. usitatus*	*F. intonsa*	
Egg period (days)	2.89 ± 0.03a	2.75 ± 0.03b	0.0090	5.03 ± 0.03a	4.34 ± 0.02b	<0.0001
Nymphal period (days)	3.94 ± 0.09a	2.96 ± 0.05b	<0.0001	9.03 ± 0.10a	7.75 ± 0.11b	<0.0001
Pupa period (days)	1.89 ± 0.08a	1.47 ± 0.05b	0.0006	3.72 ± 0.10a	2.07 ± 0.07b	<0.0001
Total preadult period (days)	8.67 ± 0.12a	7.14 ± 0.05b	<0.0001	17.58 ± 0.11a	14.50 ± 0.09b	<0.0001
Female adult longevity (days)	29.53 ± 1.44b	34.00 ± 1.38a	0.0248	45.27 ± 2.38a	45.87 ± 1.88a	0.8441
Female total longevity (days)	38.28 ± 1.43a	41.25 ± 1.38a	0.1371	62.47 ± 2.41a	60.43 ± 1.86a	0.5101
Total preoviposition period (days)	9.98 ± 0.21a	8.22 ± 0.09b	<0.0001	18.75 ± 0.19a	15.97 ± 0.16b	<0.0001
Oviposition period (*O*_*d*_) (days)	26.29 ± 0.32a	29.25 ± 1.15a	0.0721	37.83 ± 1.76a	33.03 ± 1.39b	0.0330
Fecundity (*F*) (eggs/female)	236.67 ± 15.25a	224.83 ± 11.02a	0.5305	220.80 ± 10.92a	179.83 ± 7.20b	0.0018

Values are mean ± SE; different lowercase letters in the same row indicate significant differences between the 2 thrip species under the same seasonal temperature treatment (paired bootstrap test, *B* = 100,000, *P* < 0.05).

The population demographic parameters of parthenogenetic *M. usitatus* and *F. intonsa* under summer and winter regimes are detailed in Table 2. The net reproduction rate (*R*_*0*_) and gross reproductive rate of *F. intonsa* were significantly shorter compared with *M. usitatus* in winter, but were not significantly different under a summer regime. The intrinsic rate of increase (*r*) and finite rate of increase (*λ*) of *F. intonsa* were significantly greater than that those of *M. usitatus*, and the mean generation time (*T*) of *M. usitatus* was significantly shorter than that of *F. intonsa* under both summer and winter regimes.

**Table 2.** Population parameters of parthenogenetic *Megalurothrips usitatus* and *Frankliniella intonsa* reared on cowpea pods under summer and winter regimes

Parameters	Summer		*P*-value	Winter		*P*-value
	*M. usitatus*	*F. intonsa*		*M. usitatus*	*F. intonsa*	
Net reproduction rate (*R*_*0*_) (offspring)	236.67 ± 15.25a	224.83 ± 11.02a	0.5305	220.80 ± 10.92a	179.83 ± 7.20b	0.0018
Intrinsic rate of increase (*r*) (day^−1^)	0.3473 ± 0.0084b	0.3888 ± 0.0048a	0.0002	0.1855 ± 0.0017b	0.2079 ± 0.0023a	<0.0001
Finite rate of increase (*λ*) (day^−1^)	1.4153 ± 0.0012b	1.4752 ± 0.0071a	0.0001	1.2038 ± 0.0021b	1.2311 ± 0.0028a	<0.0001
Mean generation time (*T*) (days)	15.74 ± 0.31a	13.93 ± 0.14b	<0.0001	29.10 ± 0.32a	24.97 ± 0.26b	<0.0001
Gross reproductive rate (*GRR*) (offspring/female)	267.90 ± 13.93a	244.86 ± 10.06a	0.1725	267.37 ± 11.42a	211.03 ± 7.05b	<0.0001

Values are mean ± SE; different lowercase letters in the same row indicate significant differences between the 2 thrip species under the same seasonal temperature treatment (paired bootstrap test, *B* = 100,000, *P* < 0.05).

### Age–Stage-specific Survival Rate and Age–Stage-Specific Fecundity Under Summer and Winter Regimes

**Fig. 1.**
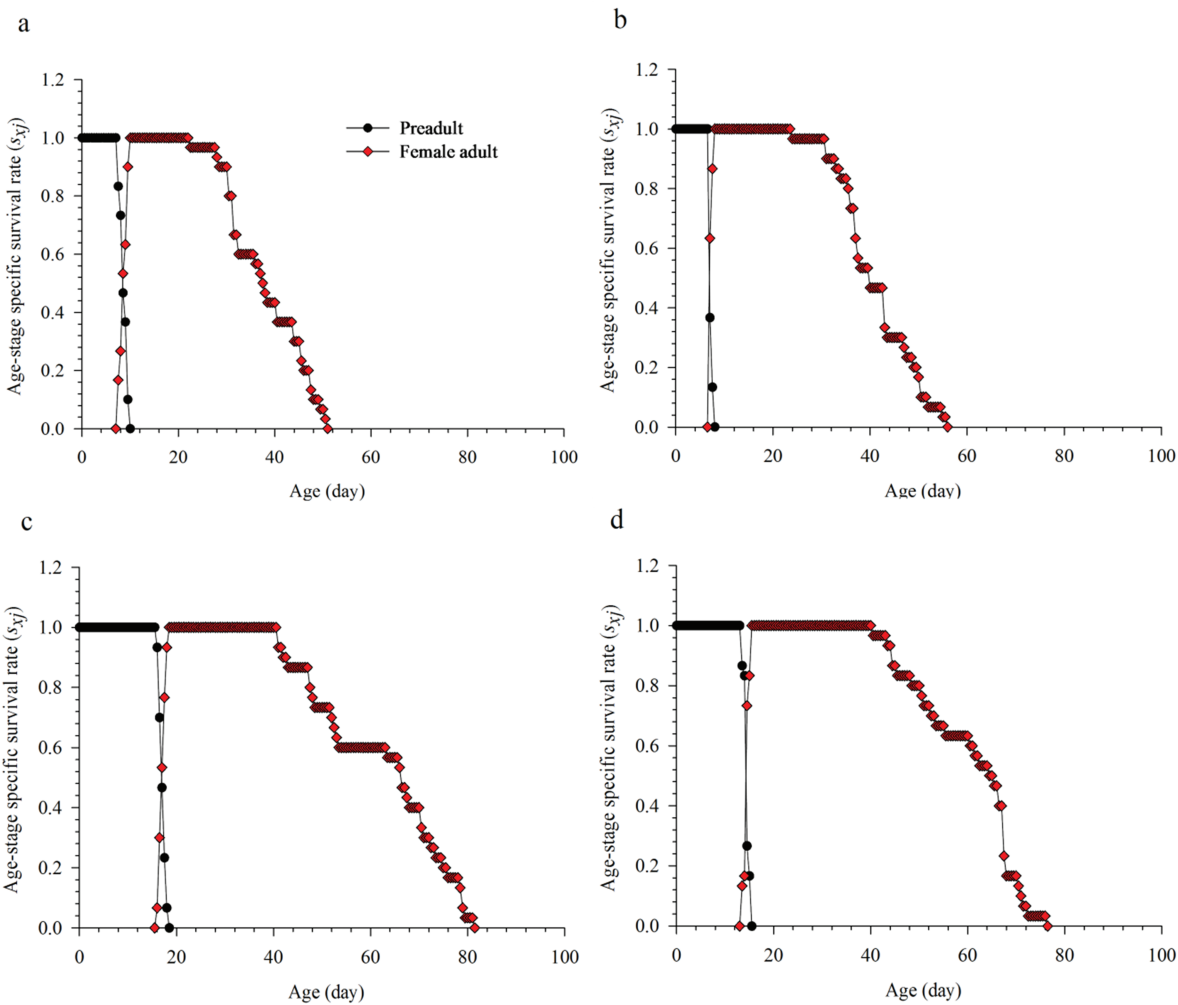
Age–stage specific survival rate (*s*_*xj*_) of *Megalurothrips usitatus* and *Frankliniella intonsa* on cowpea pods in a) summer × *M. usitatus*, b) summer × *F. intonsa*,c) winter × *M. usitatus*, and d) winter × *F. intonsa*.

The age–stage survival rates (*s*_*xj*_) of parthenogenetic *M. usitatus* and *F. intonsa* under summer and winter regimes are compared in Fig. 1, where evident overlaps can be seen between the stages. Female adult *F. intonsa* began to emerge at the age of 7 days and 13.5 days, compared with 7.5 days and 16 days for *M. usitatus*, under summer and winter regimes, respectively. In addition, adult *F. intonsa* females survived for an average of 49 days in summer, compared with 43.5 days for *M. usitatus*, with the opposite seen in winter.

**Fig. 2.**
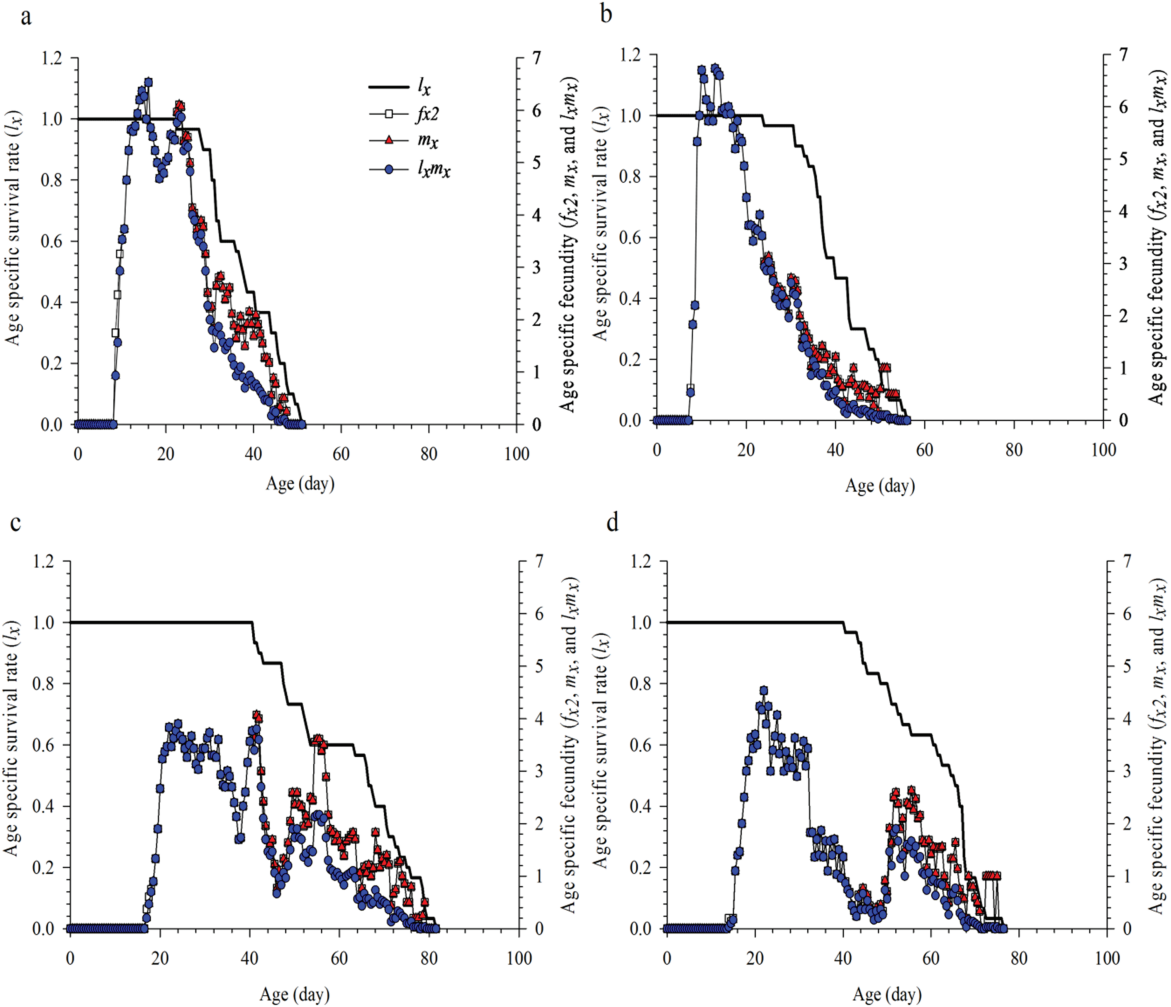
Age-specific survival rate (*l*_*x*_) and fecundities (*f*_*x2*_, *m*_*x*_, and *l*_*x*_*m*_*x*_) of *Megalurothrips usitatus* and *Frankliniella intonsa* on cowpea in a) summer × *M. usitatus*, b) summer × *F. intonsa*, c) winter × *M. usitatus*, and d) winter × *F. intonsa*.

The age-specific survival rate (*l*_*x*_), age-specific fecundity (*f*_*x2*_), age-specific fecundity of population (*m*_*x*_), and age-specific net reproductive rate (*l*_*x*_*m*_*x*_) of parthenogenetic *M. usitatus* and *F. intonsa* during the summer and winter are illustrated in Fig. 2. The *l*_*x*_ showed that the adult female *F. intonsa* began to die at the age of 24 days, which was longer than that of *M. usitatus* (22.5 days) under a summer regime, whereas *l*_*x*_ was longer in *M. usitatus* (41 days) than in *F. intonsa* (40.5 days) under a winter regime. The peak *m*_*x*_ of *M. usitatus* and *F. intonsa* was 6.53 and 6.73 at the age of 16 days and 13 days in summer and 4.07 and 4.53 at the age of 41.5 and 22 days in winter, respectively. The *m*_*x*_, *f*_*x2*_, and *l*_*x*_*m*_*x*_ curves of *M. usitatus* and *F. intonsa* showed double peaks in summer, gradually decreasing after peaking on 16 and 10 days, respectively, and then increasing again from 18.5 to 23 days or from 11.5 to 13 days, respectively, before finally decreasing again; in contrast, the curves were less pronounced under a winter regime (Fig. 2).

### Life Expectancy and Reproductive Value Under Summer and Winter Regimes

**Fig. 3.**
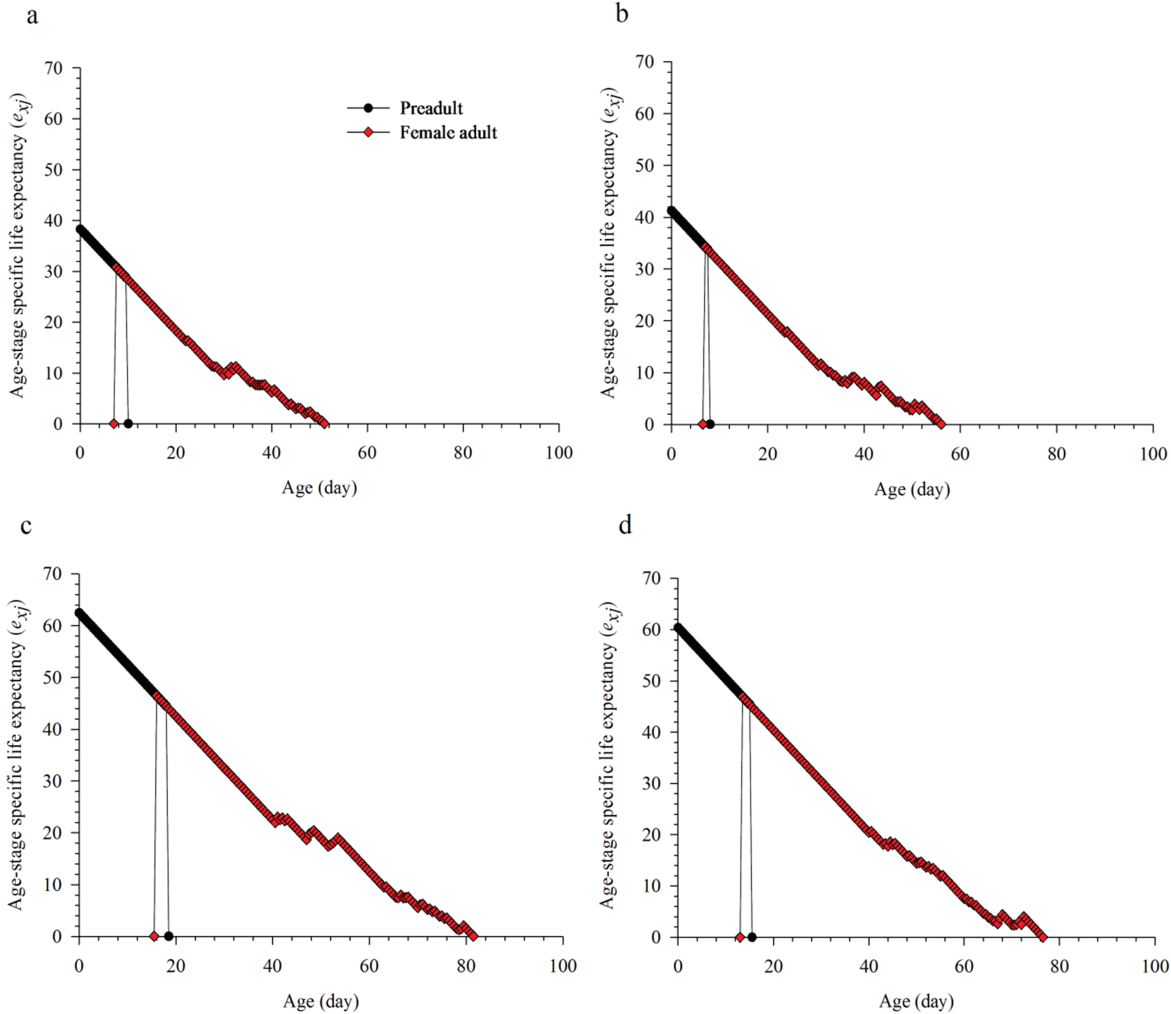
Age stage-specific life expectancy (*e*_*xj*_) of *Megalurothrips usitatus* and *Frankliniella intonsa* on cowpea in a) summer × *M. usitatus*, b) summer × *F. intonsa*, c) winter × *M. usitatus*, and d) winter × *F. intonsa*.

The life expectancy values (*e*_*xj*_) of *M. usitatus* and *F. intonsa* in winter were significantly longer than those in summer, regardless of whether newly laid eggs or adult females. The *e*_*xj*_ of a newly laid *M. usitatus* egg (38.28 days) was shorter than that of *F. intonsa* (41.25 days) in summer, but *M. usitatus* had a higher *e*_*xj*_ for newly laid eggs in winter (Fig. 3).

**Fig. 4.**
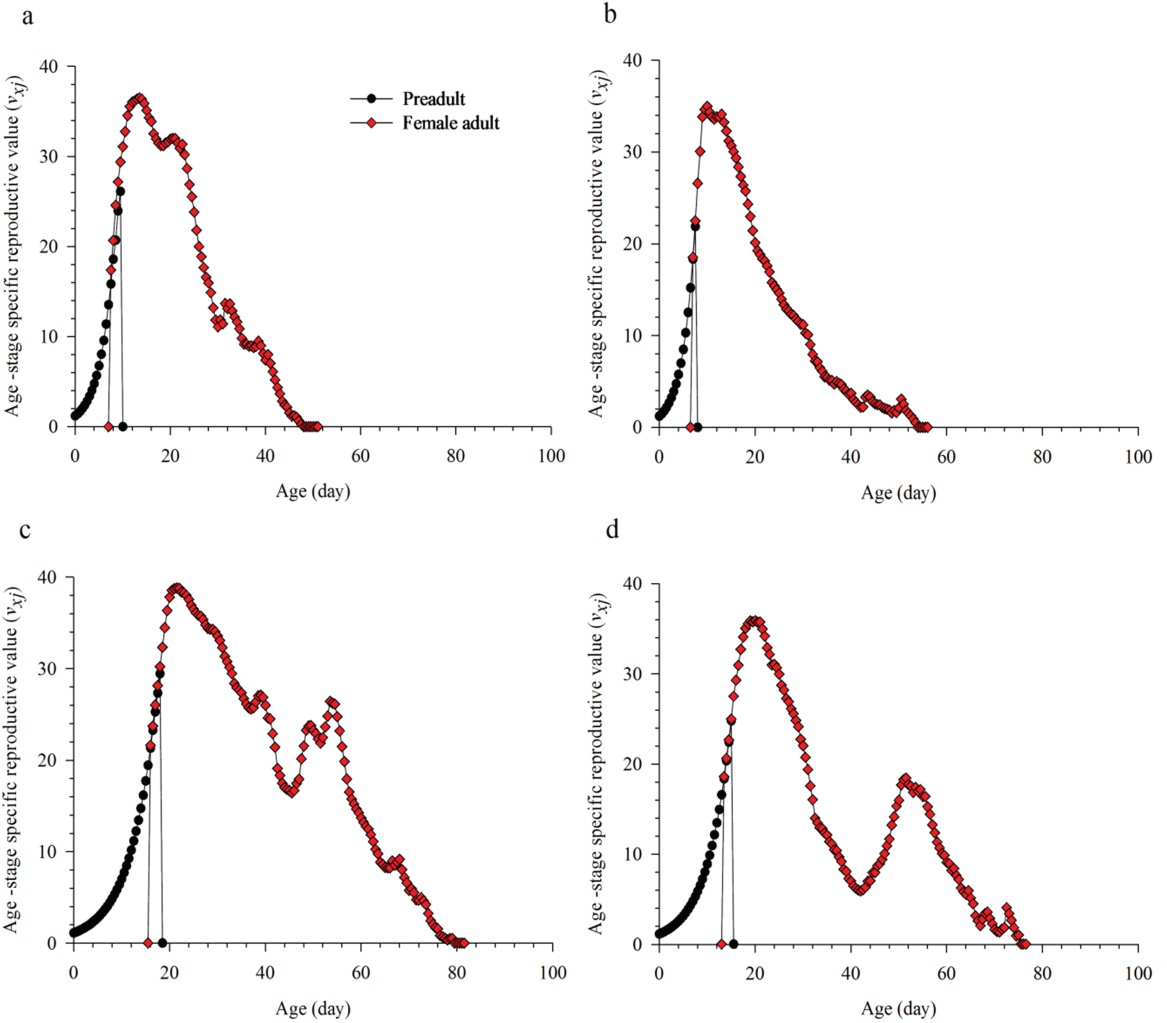
Age stage-specific reproductive value (*v*_*xj*_) of *Megalurothrips usitatus* and *Frankliniella intonsa* on cowpea in a) summer × *M. usitatus*, b) summer × *F. intonsa*, c) winter × *M. usitatus*, and d) winter × *F. intonsai*.

The reproductive value (*v*_*xj*_) of both *M. usitatus* and *F. intonsa* first gradually increased with age, peaking at 13.5 days (36.49 d^−1^) and 10 days (34.99 d^−1^) in summer and at 21.5 days (38.81 d^−1^) and 20 days (35.88 d^−1^) in winter, respectively, and then decreased with age (Fig. 4). In contrast, *v*_*xj*_ of *M. usitatus* and *F. intonsa* in winter decreased significantly to 16.42 d^−1^ and 5.88 d^−1^, respectively, and the increased to 26.42 and 18.44 d^−1^, before decreasing again (Fig. 4).

## Discussion

Interspecific competition is a natural phenomenon in the relationship between species inhabiting the same niche (Hasik et al. 2023, Wang et al. 2023). *Megalurothrips usitatus* and *F. intonsa* are 2 species inhabiting the same cowpea niche. A recent study reported interspecific competition between these species, which is thought to be driven by insecticides (Fu et al. 2022). The present study showed that some population parameters differed significantly between the 2 thrips species when in parthenogenesis under natural summer and winter regimes. The total preadult period of *F. intonsa* was significantly shorter than that of *M. usitatus* both in summer and winter. Adult females of *F. intonsa* had a longer life span with a shorter preoviposition period in summer, although female longevity was not significantly different between the species. The *R*_0_ of *F. intonsa* was significantly shorter than that of *M. usitatus* in winter, and *F. intonsa* also had higher *r* and *λ* with a lower *T* value under both summer and winter regimes. These demographic parameters suggested that *F. intonsa* had more interspecific competitive advantages compared with *M. usitatus*. Similar results were also found in *M. usitatus* and *F. intonsa* reproducing sexually (Tang et al. 2023). In addition, thrips pests are small size and usually cause economic losses to crops through aggregation damage. Fecundity is an important indicator of its economic importance. We found that the fecundity and *R*_0_ values were the same because it was not possible to estimate the survival of immature female thrips (i.e., *l*_*x*_ = 1 in the immature stage) (Zhao et al. 2021), although *M. usitatus* were significant higher than *F. intonsa* in winter regimes. This is also consistent with the results of field investigation (Tang et al. 2015a, Fu et al. 2022). Therefore, it is helpful to guide the field control.

Earlier research showed that some life table parameters change significantly when insects reproduce in different ways (Stouthamer and Luck 2011, Li et al. 2012, Tang et al. 2015b). This study found that *M. usitatus* and *F. intonsa* had a shorter developmental time and higher fecundity when reproducing parthenogenetically. The life table parameters of *M. usitatus* reproducing sexually showed that adult longevity, oviposition period, and fecundity were 14.48 days, 11.87 days, and 77.53 eggs/female, respectively (Tang et al. 2015b), and 26.20 days, 24.10 days, and 95.70 eggs/female in *F. intonsa*, respectively (Ullah and Lim 2015), which were substantially lower than those observed in this study (adult longevity > 38.28 days, oviposition periods > 26.29 days, and fecundity > 179.83 eggs/female) (Table 1). In contrast, previous studies showed a decrease in female egg production in *F. occidentalis* (Yuan et al. 2010), a longer preoviposition period in *Phenacoccus solenopsis* Tinsley (Hemiptera: Pseudococcidae) (Zhang et al. 2016), and a longer preadult period in *Echinothrips americanus* Morgan (Thysanoptera: Thripidae) (Li et al. 2012) when reproducing parthenogenetically compared with sexually. Thus, life table characteristics are specific to different species under different reproductive forms.

Under adverse environments, parthenogenesis is an important reproductive approach to maintain the stability of population dynamics (Normark and Kirkendall 2009, O’Woma et al. 2016). However, arrhenotoky parthenogenesis appears to be dispensable in normal circumstances, because thrips usually reproduce bisexually that can produce offspring with a certain male ratio. When environment worsen, the whole population could create a situation where only females remain. This might be because, the number of thrip females resulting from sexual reproduction was significantly greater than that of males, and the female lifespan was longer (Cao et al. 2018, Tang et al. 2023). The numerical disadvantage of male thrips might result in a greater risk of extinction in the face of dramatic environmental changes.

In addition, the response of adult male and female thrips to environmental stress is asymmetric. Generally, female adult thrips are more tolerant of environmental stress compared with males (Ramchandra and Chang 2014). For example, the survival rate of adult female *F. occidentalis* was significantly higher than that of male thrips at 33~43 ºC for 2 h (Li et al. 2011). After an extreme high temperature of 45 ºC for 2 h, most of the surviving adults of *F. occidentalis* were female (Wang et al. 2014). Meanwhile, both adult female *F. occidentalis* and *F. intonsa* was more adaptable compared with males to pesticide stress, although there are differences between 2 species (Hu et al. 2018, Zhang et al. 2018). Moreover, female insects are generally more tolerant of starvation than are males, such as *Drosophila melanogaster* Meigen (Gilchrist et al. 2008), *Arma chinensis* Fallou (Zhang et al. 2017), and rice planthoppers (Hirao 1979). Therefore, adult male insects are more likely to die in the face of environmental stress.

Thus, it is necessary to increase the number of males by arrhenotoky parthenogenesis to maintain the population structure. For example, *Reticulitermes aculabialis* Tsai and *Reticulitermes speratus* Kolbe (Isoptera: Rhinotermitidae) can establish and expand their population by parthenogenesis after pairing failure (Matsuura and Nishida 2001, Matsuura et al. 2009). *Brachionus calyciflorus* Pallas (Rotifera) also respond to adverse conditions by parthenogenesis to ensure population stability (Ge and Xi 2011). In general, the thrips reproductive form is a reproductive strategy that enables active adaption to environmental changes. When the environment is unfavorable for thrips, they initiate parthenogenesis to produce more male offspring, and maintain the population size by backcrossing the male offspring of parthenogenesis with their parents (Li et al. 2016).

In conclusion, the current results indicated that the life table parameters were significantly different between the 2 species of thrips under parthenogenesis reproductive form. These findings can help in better understanding the interspecific interactions between the 2 thrips species on cowpea under changing environment and in guiding for thrips control.

## Supplementary Material

toad180_suppl_Supplementary_Material
