# Supplementary material for: Parthenogenesis affects interspecific competition between Megalurothrips usitatus and Frankliniella intonsa (Thysanoptera: Thripidae) in changing environment: evidence from life table study
Source: J Econ Entomol. 2023 Oct 4;116(6):2043–51. doi: 10.1093/jee/toad180 (PMC10711564; doi:10.1093/jee/toad180)
Supplement: toad180_suppl_Supplementary_Material [file toad180_suppl_supplementary_material.docx]

Supporting information for *Journal of Economic Entomology*

**Life table comparison of *Megalurothrips usitatus* and *Frankliniella intonsa* (Thysanoptera: Thripidae) during parthenogenesis under natural regimes**

Ling-Hang Guo^1,2^ Sheng-Yong Wu^3^, Run-Na Gong^1^, Liang-De Tang^1*^

^1^ National Key Laboratory of Green Pesticide, Guizhou University, Guiyang 550025, China

^2^ School of Plant Protection, Hainan University, Haikou 570228, China

^3^ State Key Laboratory for Biology of Plant Diseases and Insect Pests, Institute of Plant Protection, Chinese Academy of Agricultural Sciences, Beijing 100193, China

* Correspondence author: tangldcatas@163.com


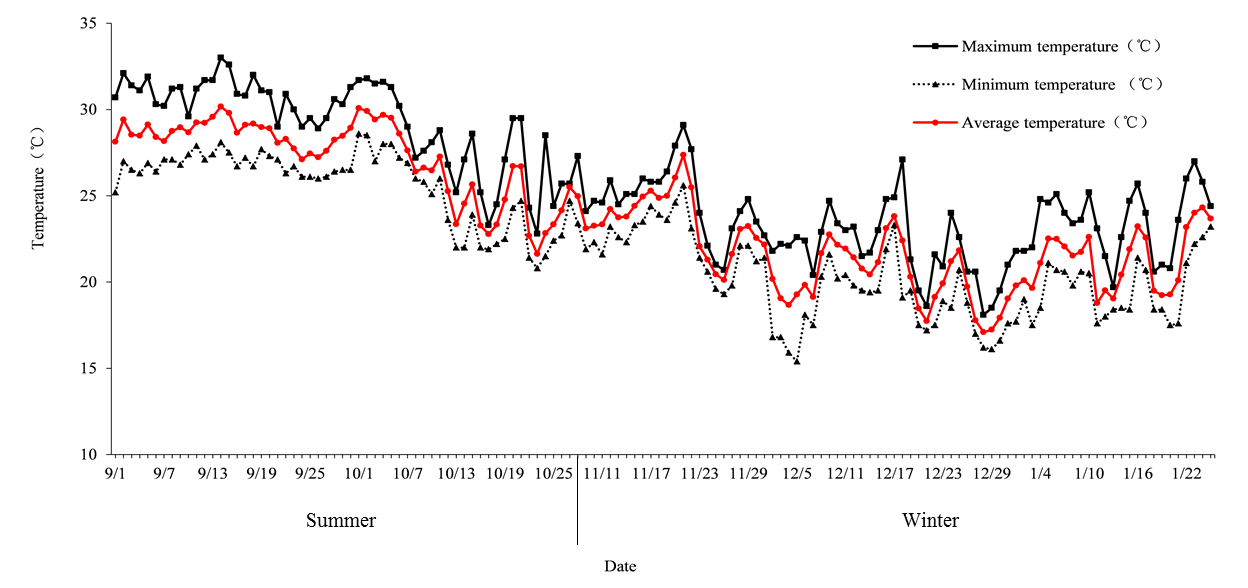


**Figure. S1.** The environmental temperature was recorded in summer (average temperature: 27.3 °C) and winter (average temperature: 21.6 °C) during the experiment in Dafeng Town, Chengmai County, Hainan Province, China. Note: Summer and winter here means most of the time is in this interval.

**Table S1. Definition and formulae of population parameters**

| Parameter | Definition and formulae |
| --- | --- |
| Age-stage survival rate (*s_xj_*) : | The probability that a newborn offspring will survive to age *x* and stage *j* and is calculated as: *s_xj_* = *n_xj_*/*n_01_*, where *n_01_* is the number of newborns at the beginning of the population and *n_xj_* is the number of individuals surviving to age *x* and stage *j*. |
| Age–stage specific fecundity (*f_xj_*): | The mean number of offspring produced by individuals at age *x* and stage *j*. |
| Age-specific survival rate (*l_x_*): | The probability that a newborn offspring survives to age *x.* It is calculated as , where *m* is the number of stages. |
| Age-specific fecundity (*m_x_*): | The mean number of offspring produced by individuals at age *x*. It is calculated as . |
| Net reproductive rate (*R_0_*): | The total number of offspring that an average individual (including females, males, and those died in immature stage) can produce during its lifetime. It is calculated as . |
| Intrinsic rate of increase (*r*): | The population growth rate as time approaches infinity and population reaches the stable age–stage distribution (SASD). The population size will increase at the rate of *e^r^* per time unit. It is calculated by using the Euler–Lotka equation with age indexed from 0: . |
| Finite rate of increase (*λ*):  . | The population growth rate as time approaches infinity and population reaches the stable age–stage distribution. The population size will increase at the rate of λ per time unit: λ = *e^r^*. |
| Mean generation time (*T*): | Period that a population requires to increase to *R_0_* -fold of its size as time approaches infinity and the population settles down to a stable age–stage distribution. *T* = ln*R*_0_/*r*. |
| Age–stage-specific life expectancy (*e_xj_*): | Time that an individual of age x and stage *j* is expected to live and is calculated by using the following equation: , where sʹ*_­iy_* is the probability that an individual of age *x* and stage *j* will survive to age *i* and stage *y* and it is calculated by assuming *s_xj_* = 1. |
| Age–stage-specific reproductive value (*v_xj_*): | Contribution of an individual of age *x* and stage *j* to the future population:. |
